# Effect of a Novel Food Rich in Miraculin on the Intestinal Microbiome of Malnourished Patients with Cancer and Dysgeusia

**DOI:** 10.3390/nu17020246

**Published:** 2025-01-10

**Authors:** Julio Plaza-Diaz, Marco Brandimonte-Hernández, Bricia López-Plaza, Francisco Javier Ruiz-Ojeda, Ana Isabel Álvarez-Mercado, Lucía Arcos-Castellanos, Jaime Feliú-Batlle, Thomas Hummel, Samara Palma-Milla, Angel Gil

**Affiliations:** 1 Department of Biochemistry and Molecular Biology II, School of Pharmacy, University of Granada, 18071 Granada, Spain; mbrandimonte@ugr.es (M.B.-H.); fruizojeda@ugr.es (F.J.R.-O.); 2 Instituto de Investigación Biosanitaria IBS.GRANADA, Complejo Hospitalario Universitario de Granada, 18014 Granada, Spain; alvarezmercado@ugr.es; 3 School of Health Sciences, Universidad Internacional de La Rioja, Avenida de la Paz, 137, 26006 Logroño, Spain; 4 Food, Nutrition and Health Platform, Hospital La Paz Institute for Health Research (IdiPAZ), 28046 Madrid, Spain; bricia.plaza@idipaz.es (B.L.-P.); lucia.arcos.castellanos@idipaz.es (L.A.-C.); samara.palma@salud.madrid.org (S.P.-M.); 5 Medicine Department, Faculty of Medicine, Complutense University of Madrid, Plaza de Ramón y Cajal, s/n, 28040 Madrid, Spain; 6 Institute of Nutrition and Food Technology “José Mataix”, Centre of Biomedical Research, University of Granada, Avda. del Conocimiento s/n. Armilla, 18016 Granada, Spain; 7 CIBEROBN (CIBER Physiopathology of Obesity and Nutrition), Instituto de Salud Carlos III, 28029 Madrid, Spain; 8 RU Adipocytes and Metabolism, Helmholtz Diabetes Center at Helmholtz Munich, German Research Center for Environmental Health GmbH Neuherberg, 85764 Neuherberg, Germany; 9 Department of Pharmacology, University of Granada, 18071 Granada, Spain; 10 Oncology Department, Hospital La Paz Institute for Health Research-IdiPAZ, Hospital Universitario La Paz, 28029 Madrid, Spain; jaime.feliu@salud.madrid.org; 11 CIBERONC (CIBER Cancer), Instituto de Salud Carlos III, 28029 Madrid, Spain; 12 Medicine Department, Faculty of Medicine, Autonomous University of Madrid, Arzobispo Morcillo 4, 28029 Madrid, Spain; 13 Smell & Taste Clinic, Department of Otorhinolaryngology, Technische Universität Dresden, Fetscherstraße 74, 01307 Dresden, Germany; thomas.hummel@tu-dresden.de; 14 Nutrition Department, Hospital University La Paz, 28046 Madrid, Spain

**Keywords:** cancer, neoplasms, dysgeusia, malnutrition, intestinal microbiome, dried miracle berries, taste disorders

## Abstract

Background/Objectives: Dysgeusia contributes to malnutrition and worsens the quality of life of patients with cancer. Despite the different strategies, there is no effective treatment for patients suffering from taste disorders provided by the pharmaceutical industry. Therefore, we developed a novel strategy for reducing side effects in cancer patients by providing a novel food supplement with the taste-modifying glycoprotein miraculin, which is approved by the European Union, as an adjuvant to medical–nutritional therapy. Methods: A pilot randomized, parallel, triple-blind, and placebo-controlled intervention clinical trial was carried out in which 31 malnourished patients with cancer and dysgeusia receiving antineoplastic treatment were randomized into three arms—standard dose of dried miracle berries (DMBs) (150 mg DMB/tablet), high dose of DMBs (300 mg DMB/tablet), or placebo (300 mg freeze-dried strawberry)—for three months. Patients consumed a DMB or placebo tablet before each main meal (breakfast, lunch, and dinner). Using stool samples from patients with cancer, we analyzed the intestinal microbiome via nanopore methodology. Results: We detected differences in the relative abundances of genera *Phocaeicola* and *Escherichia* depending on the treatment. Nevertheless, only the *Solibaculum* genus was more abundant in the standard-dose DMB group after 3 months. At the species level, *Bacteroides* sp. PHL 2737 presented a relatively low abundance in both DMB groups, whereas *Vescimonas coprocola* presented a relatively high abundance in both treatment groups after 3 months. Furthermore, a standard dose of DMB was positively associated with TNF-α levels and *Lachnoclostridium* and *Mediterraneibacter* abundances, and a high dose of DMB was negatively associated with TNF-α levels and the relative abundance of *Phocaeicola*. Following the administration of a high dose of DMB, a positive correlation was observed between erythrocyte polyunsaturated fatty acids and the presence of *Lachnoclostridium* and *Roseburia*. Additionally, a positive association was identified between *Phocaeicola* and the acetic acid concentration of feces. There was a negative association between the relative abundance of *Phocaeicola* and taste perception in the high-dose DMB group. Conclusions: The combination of DMB intake with nutritional treatment and individualized dietary guidance results in positive changes in the intestinal microbiome of patients with cancer and dysgeusia. Changes observed in the intestinal microbiome might contribute to maintaining an appropriate immune response in cancer patients. As the current pilot study included a limited number of participants, further clinical trials on a larger group of patients are needed to draw robust findings.

## 1. Introduction

Cancer is characterized by uncontrolled cell proliferation [1]. The disease affects people in many ways, including psychologically, physically, economically, and socially [2]. Many patients with cancer may benefit from systemic therapy, chemotherapy, and radiotherapy; however, these treatments are also associated with a high risk of serious complications [3].

Malnutrition is estimated to be responsible for the death of 10–20% of patients with cancer [4]. However, nutritional support is received by only 30–60% of cancer patients who are at risk of malnutrition [4].

Despite possible adverse consequences, taste changes experienced by patients with cancer are not usually diagnosed and treated early because clinicians do not consider them life-threatening [5,6,7]. It is estimated that 45 to 80% of patients experience chemotherapy-induced taste changes [8,9,10]. Dysgeusia is the umbrella term for qualitative and quantitative taste dysfunction, and includes taste distortions with bitter, metallic, salty, or unpleasant tastes [11,12,13]. The consequences of taste alterations are the deterioration of nutritional status, a reduction in quality of life, weight loss, and ultimately, health deterioration [14,15,16,17]. Zinc, amifostine, selenium, lactoferrin, and cannabinoids are currently used to treat taste disorders; however, their effectiveness is limited [18,19,20].

The gut microbiome plays a crucial role in maintaining health, influencing not only the gastrointestinal tract but also distant organs such as the brain, liver, and pancreas [21,22]. The composition of the gut microbiome is diverse: it is composed of more than 200 bacterial species [23,24] (including phylotypes such as *Bacillota*, *Bacteroidota*, *Actinomyces*, *Fusobacterium*, *Pseudomonadota*, and *Verrucomicrobiota*) [25], fungi (*Candida albicans*), viruses, and protists [26]. Microorganisms that belong to a separate kingdom of living organisms, *Archaebacteria*, are also an important part of the intestinal microbiome [27]. An alteration in the equilibrium of the gut microbiome can result in the development of a dysbiotic state, with subsequent implications for both local and systemic health outcomes [28]. Thus, dysbiosis contributes to a variety of pathologies, including obesity [29], diabetes [30], neurodegenerative diseases [31], and cancer [32]. Approximately 20% of all cancers are strongly associated with specific viral or microbial infections [33]. Furthermore, bacteria have been identified as key factors in the progression of several types of cancer, including oral squamous cell carcinoma, colorectal cancer, and pancreatic ductal adenocarcinoma [34,35,36].

The complexity of the gut microbiome, as well as its richness and abundance, predicts the metabolic health of the host [37]. Several factors contribute to the composition of the gut microbiome, including diet and dietary habits. Unsurprisingly, the gut microbiome has been associated with several cancer determinants, such as taste perception, which influences appetite regulation and energy metabolism [37]. Furthermore, there is evidence that the gut microbiome can affect the response to systemic cancer therapy [38].

Miraculin is a glycoprotein obtained from *Synsepalum dulcificum* berries that converts a sour taste into a sweet taste, which is why the fruit is also called the “miracle berry” [39]. The taste-modifying effect of miraculin occurs under acidic conditions and lasts for approximately 30 min after consumption. Two small non-randomized studies using non-objective tools tried to evaluate the effect of the miracle fruit on taste disorders in patients with cancer who were receiving active chemotherapy treatment, describing promising results [15,40]. The European Food Safety Authority (EFSA) approved dried miracle berries (DMBs) as a novel food in December 2021. In addition to their taste-modifying properties, DMBs also contain bioactive ingredients, such as fiber and phenolic compounds [41,42].

The DMB is well known for its ability to turn sour foods into sweet ones as a result of the action of the taste receptors [43,44]. The antioxidant properties of DMBs are attributed to their containment of terpenoids, phenolic compounds, and flavonoids [39]. A variety of polar extracts have been demonstrated to inhibit the proliferation and transformation of cancer cell lines in vitro [45,46]. According to a study, the DMB contains a natural inhibitor of dipeptidyl-peptidase 4 that may be useful for treating type 2 diabetes [47]. The potential of miraculin lies in its ability to act both as a natural non-caloric sweetener and as a TAS1R agonist that modulates the release of distinct enteric hormones at the gastrointestinal level [39,48]. Several authors have hypothesized that miraculin’s actions are the result of a relationship between the gut microbiome and the brain via the gut–brain axis [39]. Despite this, no studies have been conducted to evaluate the aforementioned relationship.

In a pilot randomized, parallel, triple-blind, and placebo-controlled clinical trial (the CLINMIR study), our research group provided clinical evidence on the efficacy of DMBs in improving taste alterations in cancer patients. As a result of this study, we observed improvements in electrochemical food perception, energy and nutrient intake, nutritional status, and quality of life for malnourished patients with cancer receiving antineoplastic treatment [49]. Moreover, we showed that regular DMB consumption and nutritional interventions changed the oral microbiome in patients with cancer and dysgeusia, which may contribute to maintaining an appropriate immune response without altering taste perception [50].

The purpose of the present study was to assess the intestinal microbiome of malnourished patients with cancer and dysgeusia after DMB consumption as a medical–nutritional adjuvant treatment.

## 2. Materials and Methods

### 2.1. Statement of Ethical Principles

This project was approved by University Hospital La Paz’s (HULP Code 6164) Scientific Research and Ethics Committee in June 2022. According to the Declaration of Helsinki’s Ethical Standards, this study adheres to recommendations for physicians conducting biomedical research on humans. The ICH Harmonized Tripartite Guidelines should be familiarized and followed by all researchers to maintain good clinical practices.

The research team informed the patients (verbally and in writing) of the study characteristics and the responsibilities of participation in the trial before they signed the informed consent form. Patients were informed during the study that they could withdraw from the study at any time by notifying their doctor without giving a reason. The processing of personal information is subject to several legal requirements, including Spanish Organic Law 3/2018 of 5 December and the General Data Protection Regulation of the European Union (EU) 2016/679 of 27 April 2016.

### 2.2. Participants and Experimental Design

Detailed information on the CLINMIR study is published elsewhere [49,51]. Briefly, the CLINMIR study is a pilot randomized, parallel, triple-blind, and placebo-controlled clinical trial. Using the number NCT05486260, the present protocol was registered at http://clinicaltrials.gov, accessed on 14 March 2024. An oncology service and clinical nutrition unit at HULP in Madrid recruited 31 malnourished cancer patients with taste disorders.

Three treatment arms were randomly assigned to malnourished patients with cancer and taste disorders who were receiving active treatment. A miraculin-based food supplement was administered to patients five minutes before each meal (breakfast, lunch, and dinner) during a three-month study. The tablets contained either the DMB at one of its two dosages or a placebo [49,50].

Each intervention group consisted of ten patients who were randomly assigned to receive one of two DMB dosages or a placebo. In the first arm of the study, 150 mg of DMB equivalent to 2.8 mg of miraculin was combined with 150 mg of freeze-dried strawberries; in the second arm, 300 mg of DMB was utilized, equivalent to 5.6 mg of miraculin; and in the third arm, 300 mg of freeze-dried strawberries was used as a placebo. Each of the three treatments was isocaloric (Table S1). The subjects received as many tablets as necessary during scheduled visits to the HULP to complete the three-month intervention period [49,50]. In Table S2, the types of cancer, demographics, and chemotherapy characteristics of the study population are summarized.

It was previously stated that DMBs had been approved by the EFSA as a novel food. According to the panel, an intake of 10 mg/kg body weight per day is safe for human consumption [52]. The maximum dose used in this clinical trial was 0.9 g/day, which is slightly above the recommended dosage. According to the EFSA, a 90-day oral dose of 2000 mg/kg body weight per day was not associated with adverse effects [52]. In addition, miraculin has been evaluated for its potential allergenicity and toxicity, and it is safe [53].

We determined the sample size based on the exploratory nature of the study [54,55], following international recommendations for good practice in pilot studies, and the lack of previous studies using miraculin-based supplements in cancer patients. To assess the validity of the results of this pilot study, statistical power was determined at the end of the study [49,50].

### 2.3. Sequencing of Biological Samples

To prepare for the analyses, sterile plastic containers were used to collect fecal samples at baseline and 3 months after intervention. Blood samples were collected by trained personnel at the HULP Extraction Unit in the morning (approximately at 8:00 a.m.) during blood tests before chemotherapy to avoid unnecessary punctures and hospitalizations. The blood samples were collected in vacuum tubes, labeled, transported, and centrifuged at 1500× *g* for 10 min. We prepared and labeled aliquots of blood samples according to a numerical code and stored them at −80 °C.

#### 2.3.1. Extraction of DNA

A QIAamp Fast DNA Stool Mini kit (ref. ID: 51604, Qiagen Inc., Hilden, Germany) was used to extract DNA from the stool samples. The purity and integrity of DNA were determined using spectrophotometry (NanoDrop, Thermo Fisher Scientific, Waltham, MA, USA).

#### 2.3.2. 16S rRNA Gene Sequencing and Taxonomic Assignment

A detailed description of 16S rRNA bacterial gene sequencing via Oxford Nanopore Technologies can be found elsewhere [50]. Briefly, the 16S rRNA gene was PCR-amplified using redesigned 16S primers (27F and 1492R) with 5′ tags that facilitate ligase-free attachment. By vortexing 30 μL of AMPure XP beads (Beckman Coulter, ThermoScientific, Spain) and mixing by pipetting, PCR products from each sample were cleaned. To achieve a concentration of 50–100 fmoles, all barcoded libraries were combined in the appropriate ratios. SpotON Flow Cell Mk R9 Version (ref. FLO-MIN106D, Oxford Nanopore Technologies, Oxford, UK) was used to sequence the final library using Minion M1kc and M1kb sequencers (Oxford Nanopore Technologies, Oxford, UK).

After the raw data had been generated, searches were performed via Guppy version 6.5.7 (Oxford Nanopore Technologies, Oxford, UK), and sequences were identified using Kraken2 (refseq Archaea, bacteria, viral, plasmid, human, UniVec_Core, protozoa, fungi and plant database) and further analyzed using QIIME2, a microbiome multi-omics bioinformatics and data science platform [56]. Assigning taxonomy to ASVs was performed using the classify sklearn naïve Bayes taxonomy classifier (via q2-feature-classifier) [57] using SILVA 16S V3-V4 v132_99 [58] with a similarity threshold of 99%. The diversity of the samples was examined using the vegan library [59]. In this study, Shannon, Simpson, and Chao1 indices were examined.

### 2.4. Plasma Cytokines and Biochemical Parameters

Plasma tumor necrosis factor-alpha (TNF-α) and human proteolysis-inducing factor (PIF) were analyzed as previously described [60]. The Biochemistry Laboratory at the Hospital La Paz, an ISO-certified laboratory, performed biochemical analyses using an Olympus AU5400 Automated Chemistry Analyzer (Olympus Corporation, Izasa, CA, USA) [49,51]. A summary of key blood cells and biochemical parameters is provided in Table S3.

### 2.5. Dietary Pattern Assessment

For three days, including one holiday, daily food records were kept. Patients were advised to record household measurements (spoonfuls, cups, etc.) or household weights in the absence of weight records. A nutritionist reviewed all records in the presence of the patient to ensure that the information collected was accurate and complete. DIAL software (Alce Ingeniera, Madrid, Spain) was used to convert the energy and nutrients contained in foods, drinks, dietary supplements, and preparations. Finally, the results were compared with the recommended intakes for the Spanish population [49,50].

### 2.6. Short-Chain Fatty Acid Determination by Gas Chromatography/Mass Spectrometry

A total of 100 μL of plasma was individually placed in 1.5 mL tubes. Afterward, 10 μL of acidified water (15% phosphoric acid *v*/*v*) and 10 μL of internal standards (sodium acetate ^13^C2 at 300 μM, butyric-1,2-^13^C2 at 60 μM, and isobutyric acid d6, valeric acid d9, isovaleric acid d9, and propionic d6 acid at 30 μM) were added and vigorously mixed. Next, a liquid–liquid extraction was performed with 150 μL of MTBE. The extraction was assisted by vortexing for 10 min. At this point, tubes were centrifuged at 15,000 rpm for 10 min at 4 °C. Then, 100 μL was transferred into a vial with an insert. The vials were centrifuged at 1000 rpm for 30 s at 4 °C, and 1 μL was injected into the gas chromatography/mass spectrometry apparatus. Briefly, short-chain fatty acids were separated on a DB-FFAP chromatographic column (30 m × 0.25 mm × 0.25 μm). The oven temperature was programmed as follows: (i) initial temperature 40 °C, (ii) linearly increased at 12 °C/min until 130 °C (0 min), (iii) then linearly raised at 30 °C/min to 200 °C (0 min), and (iv) in the final step, the temperature was ramped at 100 °C/min to 250 °C (4.5 min). The column flow was set at 1.5 mL/min with helium as the carrier gas. The injector was set at 250 °C and the extract was injected in split-less mode. Using electronic impact (70 eV) for ionization, the mass analyzer was operated for multi-reaction monitoring.

### 2.7. Electrical Taste Perception

Taste perception was evaluated using electrogustometry. To quantify human taste perception objectively, electrical taste testing is an excellent method [61]. Functional imaging studies have shown that lingual electrical stimulation activates the same brain regions as chemical stimulation [62]. Patients with cancer and taste distortion and who consume miraculin-based food supplements are expected to improve their taste perception by reducing their taste perception threshold (measured in decibels, dB) via electrical stimulation at baseline, one month after the intervention with DMB, and three months thereafter, as measured by electrical stimulation [49,50,51]. An electrogustometer (SI-03 Model, Sensonics International, Haddon Heights, NJ, USA) was used to measure the threshold for an electrically induced taste stimulus. An electrode is placed on the tongue to apply the electric stimulus. To familiarize the patient with the electrical stimulus, a first stimulus (30 dB) is administered. Upon determining the threshold, stimulation begins at the zero-stimulus amplitude and increases progressively until the patient identifies the stimulus. A stimulus-response staircase and the two-down one-up forced-choice single staircase were used to measure detection thresholds [49,50,51].

### 2.8. Statistical Analysis

To examine the effects of time, treatment, and their interaction (time × treatment), a general linear mixed model of covariance was used to examine the differences between placebo, 150 mg of DMB, and 300 mg of DMB using cancer treatment success as a covariate. Using the R 4.4.2 program, a general linear mixed model was developed using the lme4 package [63]. A median test revealed significant differences across time points within groups.

We also examined the relationships between intestinal microbiome variables, inflammatory parameters, dietary variables, short-chain fatty acids, and electrical taste perception outcomes via Pearson’s correlations; for that purpose, we used R Studio’s corrplot function [64] correcting multiple testing using the FDR procedure [65]. Only significant and corrected associations are shown in the graphs. The red and blue lines in the graphs indicate correlation values, with negative correlations highlighted in red (−1) and positive correlations highlighted in blue (+1).

Rivera-Pinto analysis can identify microbial signatures, i.e., groups of microbes capable of predicting particular phenotypes of interest. This microbial signature may be used to diagnose, prognosticate, or predict therapeutic response on the basis of the unique microbiome of an individual. Identifying microbial signatures requires modeling the response variable and selecting the taxa that are the most accurate at classification or prediction. To select a sparse model that adequately explains the response variable, we evaluated specific signatures at the phylum and genus levels using the Rivera-Pinto method and the Selbal algorithm. Based on data collected from two groups of taxa, microbial signatures were calculated using geometric means. These groups are those with relative abundances or balances that are related to the response variable of interest [66].

## 3. Results

From November 2022 to May 2023, 62 patients were assessed for eligibility. Among 31 patients with cancer who met the inclusion criteria, three intervention groups were randomly assigned according to the type of cancer. In the course of the study, ten participants withdrew from the study. Several of these dropouts were caused by taste distortions caused by acidic foods that were not sweet (n = 6) and the complexity of the intervention prescription (n = 2). During the course of the study, two placebo patients died. A total of 21 patients with cancer completed the clinical trial. All variables were analyzed with an intention-to-treat approach. The sample consisted of 58.1% women and 41.9% men, with an average age of 60.0 ± 10.9 years. Participants who were actively treated were assessed by electrogustometry; results of taste perception for the population have been published elsewhere [51].

### 3.1. Phylum Level

At baseline, *Bacillota* and *Bacteroidota* accounted for more than 80% of the relative abundance of the intestinal microbiome. Based on the comparison between baseline and three months, no major differences were found among the groups. According to the treatment, only *Pseudomonadota* was significantly different among the three study groups. Both the alpha diversity indices (Shannon, Simpson, and Chao1) and the studied phyla did not show any effect of treatment × time (Table 1).

### 3.2. Genus Level

The most common genus in all the studied groups was *Faecalibacterium* (approximately 11 to almost 20% relative abundance). There were differences between the genera *Phocaeicola* and *Escherichia* depending on the treatment. For *Solibaculum*, we observed significant differences in the interaction effect of treatment × time. The standard dose of DMB produced a significant increase in the relative abundance of *Solibaculum*, whereas the placebo resulted in a significant decrease in the relative abundance of this genus (Table 2).

### 3.3. Species Level

Four species dominated the intestinal microbiome of cancer patients: *Faecalibacterium prausnitzii*, *Anaerobutyricum hallii*, *Vescimonas coprocola,* and *Vescimonas fastidiosa*. For *Bacteroides* sp. PHL 2737 and *Vescimonas coprocola*, we observed significant differences in the interaction between treatment and time; between baseline and 3 months, *Bacteroides* sp. PHL 2737 decreased significantly in both the DMB groups and *Vescimonas coprocola* decreased in the placebo group (Table 3).

### 3.4. Short-Chain Fatty Acids

In all the study groups, acetic acid was the most abundant short-chain fatty acid. For acetic acid, there were significant differences between times and the interaction of treatment and time. As a result of treatment with the standard dose of DMB, the acetic acid level increased significantly, whereas the level decreased in patients receiving the placebo treatment (Table 4).

### 3.5. Rivera-Pinto Test for Microbiome Balance

**Figure 1:**
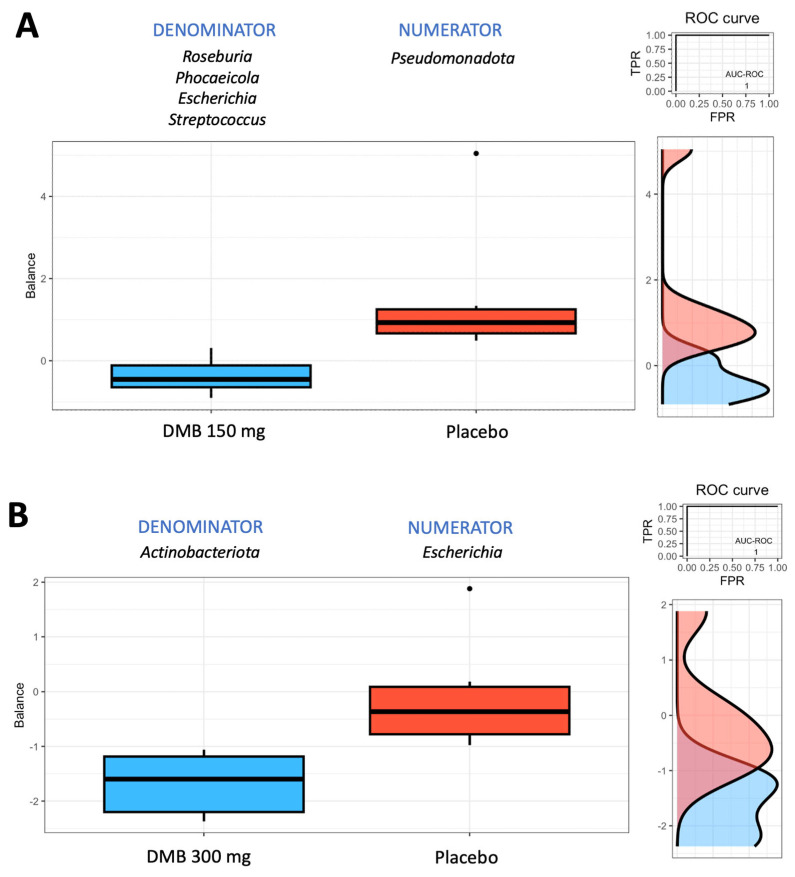
Group balances are presented in an overview. The top of the plot indicates that groups of taxa constitute the global balance. Box plots illustrating the distribution of balance scores for the DMB 150 mg (standard dose) and placebo groups (**A**) and the DMB 300 mg (high dose) and placebo groups (**B**). On the right, the ROC curve with its AUC value and the density curve are displayed.

To ascertain the microbiome balance at the conclusion of the trial, the Rivera-Pinto method was employed [66]. The analysis revealed that *Pseudomonadota* was most associated with the placebo group when the standard-dose DMB group (150 mg) was compared with the placebo group (Figure 1A). In the standard-dose DMB group, lower balance scores were associated with lower relative abundances of *Roseburia, Phocaeicola, Escherichia,* and *Streptococcus* than *Pseudomonadota* (Figure 1A). With respect to the high-dose DMB group versus the placebo group, *Escherichia* was the most strongly associated with the placebo group (Figure 1B). Thus, the higher the dose of DMB was, the lower the balance scores associated with lower relative abundances of *Actinobacteriota* than those of *Escherichia* (Figure 1B).

### 3.6. Analysis of the Relationships Among the Intestinal Microbiome, Nutritional Status, Electrical Taste Perception Inflammatory Cytokines, and Plasma Short-Chain Fatty Acids

**Figure 2:**
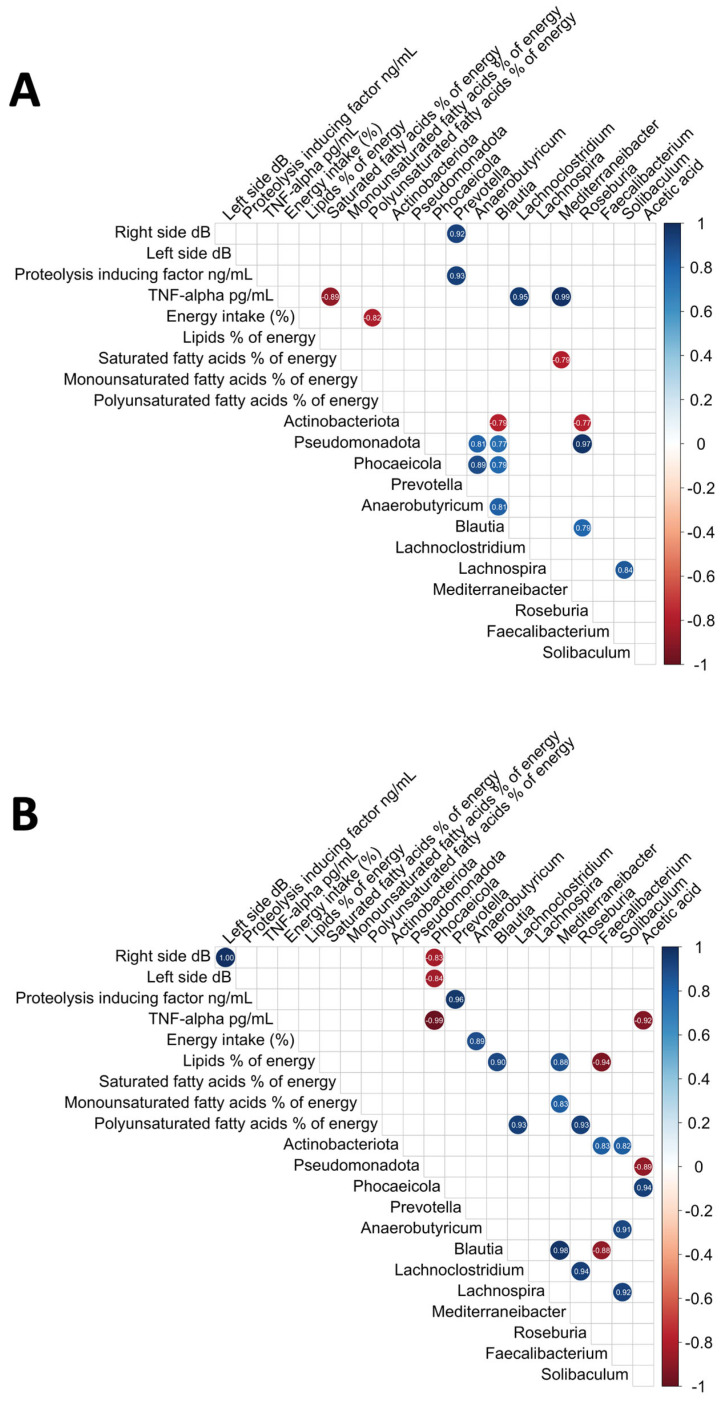

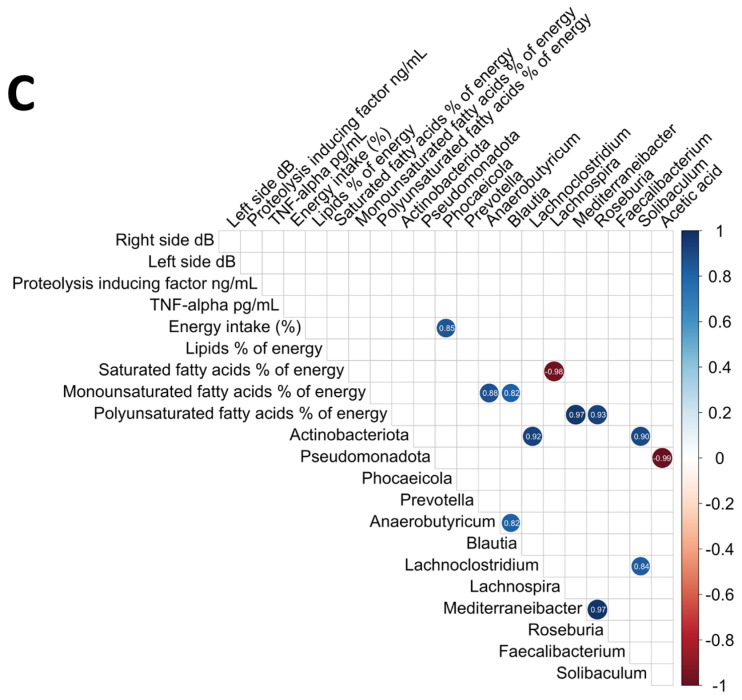
Correlations between the intestinal microbiome, nutritional status, electrical taste perception, and inflammatory cytokines. (**A**) DMB 150 mg (standard dose), (**B**) DMB 300 mg (high dose), and (**C**) placebo.

In the group of patients with cancer and dysgeusia who received the standard dose of DMB, *Mediterraneibacter* had a negative correlation with the saturated fatty acid percentage of energy in the diet. TNF-α levels were positively correlated with *Lachnoclostridium* and *Mediterraneibacter*. The presence of the *Prevotella* genus was positively correlated with the electrogustometry values on the right side of the tongue and the PIF (Figure 2A).

Several correlations were observed in the group that received high doses of DMB (Figure 2B). As a percentage of energy, *Blautia* and *Mediterraneibacter* were positively associated with lipids in the diet, whereas *Faecalibacterium* was negatively associated. *Mediterraneibacter* was positively correlated with dietary monounsaturated fatty acids. There was a positive correlation between dietary polyunsaturated fatty acids (PUFAs) and *Lachnoclostridium* and *Roseburia*. Dietary energy intake (%) was positively correlated with *Anaerobutyricum*. There was a negative correlation between the relative abundance of *Phocaeicola* and electrogustometry (both right and left sides of the tongue) and TNF-α levels. The PIF was positively correlated with the *Prevotella* genus. There was a positive correlation between *Phocaeicola* and plasma acetic acid concentration, whereas a negative correlation was detected between *Pseudomonadota* and acetic acid levels (Figure 2B).

A positive association between *Phocaeicola* and energy intake was detected in the placebo group. Dietary saturated fatty acids (%) were negatively associated with *Lachnospira*. Dietary monounsaturated fatty acids were positively correlated with *Anaerobutyricum* and *Blautia*. Dietary PUFAs were positively correlated with *Mediterraneibacter* and *Roseburia* (Figure 2C).

## 4. Discussion

The present study revealed that regular DMB consumption together with nutritional treatment and individualized dietary advice changed the composition of the gut microbiome. The major findings of the present study were that the intervention with a standard dose of DMB led to relevant changes in the intestinal microbiota, i.e., increases in the relative levels of the *Solibaculum* genus, *Bacteroides* sp. PHL 2737, and *Vescimonas coprocola* and a concomitant production of acetic acid. In addition, the *Prevotella* genus was positively associated with taste perception. In addition, selected bacteria were correlated with the intake of total energy and dietary saturated and polyunsaturated fatty acids. Furthermore, the abundance of some bacteria such as *Phocaeicola* was negatively associated with the proinflammatory cytokine TNF-α levels, and the PIF plasma levels were positively correlated with the *Prevotella* genus. Those results may be due to the higher intake of energy derived from an amelioration in the food dietary pattern promoted by miraculin intervention. Indeed, we have previously documented that cancer patients in the present study increased their intake of energy mainly at the expense of fat, which in turn led to a better status of polyunsaturated fatty acids.

Taste is especially important when dealing with diseases such as cancer, where chemotherapy may alter taste perception (dysgeusia) [67]. Food consumption has the potential to modify the intestinal microbiome and directly impacts the microbes present in the gut. As far as we know, this is the first pilot study to evaluate DMBs’ effects on the intestinal microbiome. A recent study examined the gut microbiome of patients with severe mucositis, which differed from our patients with grade 1–2 mucositis, showing an increase in the abundances of *Mediterraneibacter (Ruminococcus gnavus*) and *Clostridiaceae*, including *Hungatella hathewayi* [68]. In our study, the habitual consumption of a standard dose of DMB was positively associated with TNF-α levels and *Lachnoclostridium* and *Mediterraneibacter* abundances, whereas a high dose of DMB was negatively associated with TNF-α levels and the relative abundance of *Phocaeicola*. There has been some evidence that microbes, along with metabolites derived from gut bacteria, pathogen-associated molecular patterns, and antigens, could move from the gastrointestinal tract to other closely related tissues, which can influence the progression of cancer [69,70].

Species from *Bacteroides* and *Phocaeicola* genera play crucial roles in the human colon. By degrading complex heteropolysaccharides into short-chain fatty acids, those organisms contribute to the body’s use of these compounds [71]. We found that DMB consumption at high doses was positively associated with the abundance of the genus *Phocaeicola* and acetic acid concentrations. In addition, following DMB administration at a high dose, a positive association was found between PUFAs, *Lachnoclostridium*, and *Roseburia*. We have previously reported that the intervention with DMBs results in a better status of PUFAs, as measured in red blood cells, derived from an improved intake of nutrients [51]. PUFAs have antitumor activity; in particular, it has been proposed that PUFAs, specifically eicosapentaenoic acid and docosahexaenoic acid, possess anti-colorectal cancer activity [72]. A recent study investigated the impact of PUFA supplementation on the fecal microbiome in middle-aged, healthy volunteers, showing that PUFA supplementation leads to a reversible increase in bacteria that produce short-chain fatty acids [73]. Moreover, a reversible increase in the abundance of several bacterial genera, including *Bifidobacterium*, *Roseburia,* and *Lactobacillus*, was observed in patients who received one or both PUFA treatments. Consequently, DMB may enhance the presence of microorganisms involved in SCFA production and contribute to dietary PUFA consumption.

Numerous diseases in humans have been associated with changes in the gut microbiome composition, with fluctuations in the prevalence of particular bacterial groups. In this regard, *Faecalibacterium* was one of the most notable genera in our study. The relative abundance of this bacteria was estimated to be between 11% and 20%. A recent study revealed a negative correlation between the abundance of *Faecalibacterium* and increased intra-individual variability in microbiome composition, indicating that it is a keystone taxon [74,75]. Certain species of *Faecalibacterium* have been observed to undergo alterations in several diseases and disorders. In fact, multiple studies have demonstrated that a high baseline level of *Faecalibacterium*, along with that of other *Bacillota*, is positively correlated with responses to related treatments for various cancers, including melanoma [76,77,78,79,80], hepatocellular carcinoma [81] and non-small-cell lung cancer [82]. As a result of DMB intervention, we observed a tendency to decrease the abundance of *Faecalibacterium prausnitzii* in DMB groups.

We showed that a standard dose of DMB administration resulted in a significant increase in the relative abundance of *Solibaculum* genus, whereas placebo resulted in a reduction in this genus, which participates in pyrimidine metabolism [83]. *Bacteroides* sp. PHL 2737 and *Vescimonas coprocola* showed significant differences between the studied groups. A significant decrease in *Bacteroides* sp. PHL 2737 levels was observed between the baseline and the three months in both the DMB groups. Additionally, *Vescimonas coprocola* levels decreased significantly in the placebo group. Environmental alterations caused by dysbiosis can result in a gradual decline in functional redundancy, either as a consequence of disease or its treatment. A recent study demonstrated that colorectal cancer is influenced by the co-occurrence of species, including *Vescimonas coprocola* and *Vescimonas fastidiosa*, although they did not significantly differ in abundance [84].

Metagenomic studies based on colorectal cancer datasets have reported an association between specific microbial species and this type of cancer [85,86,87,88,89,90,91,92,93]. For example, multiple studies have demonstrated the main role of particular species in the development of colorectal cancer [94], such as *Streptococcus gallolyticus* [95], *Bacteroides fragilis* [96,97], and *Fusobacterium nucleatum* [98,99,100]. Moreover, it has been proposed that *Bacteroides fragilis* [96,97] and *Fusobacterium nucleatum* [98,99,100] are key factors in the tumorigenesis process, and then they may be replaced by “passenger” species that are favored by the cancer microenvironment [101]. In our study, DMB intake together with nutritional treatment and individualized dietary advice resulted in changes in the intestinal microbiome of cancer patients and patients with dysgeusia, which were associated with taste perception in the high-dose DMB group. Following DMB administration, patients could experience better food taste and improve their diet, as well as their food intake. Indeed, this dietary change is reflected in microbiota alterations and a better quality of life.

A possible mechanism of action of DMB and its effects on the intestinal microbiome may be mediated by the production of acetic acid, the main short-chain fatty acid that was increased following treatment with DMB. In addition, it is important to note that this is the first pilot study that has evaluated the effects of DMB on the intestinal microbiome, and comparisons are difficult. Nevertheless, the changes were correlated in a significant proportion with clinical and biochemical parameters in DMB treatments.

Through the use of novel therapies and strategies, patients with cancer can maintain their nutritional intake and enjoy their meals while experiencing changes in taste perception and aftertaste [102]. It is, however, important for patients to discuss their dietary preferences and modifications with healthcare professionals to ensure that they receive adequate nutrition while undergoing cancer treatment [102]. The ability to manipulate gut microbiome composition to improve cancer therapy outcomes is a significant new area of research [103,104]. Intestinal microbiome composition is susceptible to changes due to diet and the environment, so educating patients on food consumption during cancer treatment and avoiding carcinogens may improve outcomes [103,104].

Cancer patients receiving a standard dose or a higher dose of DMB experienced different changes in their gut microbiome from those receiving a placebo. The difference could be attributed to the sweet taste experienced following the ingestion of orodispersible DMB tablets before each main meal, compared to the placebo group, which may lead to a better dietary intake.

## 5. Conclusions

This pilot randomized, parallel, triple-blind, and placebo-controlled clinical trial identified a putative innovative aid for the management of taste disorders in patients with cancer. This novel strategy was designed with the intent of reducing the adverse effects associated with chemotherapeutic, radiotherapeutic, and immunotherapeutic interventions, which may include alterations in taste, changes in body composition and nutritional status, and alterations in quality of life [51]. Here, we observed differences between the genera *Phocaeicola* and *Escherichia* depending on the treatment. Only the *Solibaculum* genus increased in relative abundance in the DMB group after 3 months. Concerning species, *Bacteroides* sp. PHL 2737 had a lower relative abundance in both DMB groups, and *Vescimonas coprocola* exhibited a greater abundance in both treatments after 3 months. Moreover, a standard dose of DMB was positively associated with TNF-α levels and *Lachnoclostridium* and *Mediterraneibacter* abundances, whereas a high dose of DMB was negatively associated with TNF-α levels and the relative abundance of *Phocaeicola*. After high-dose DMB administration, a positive correlation was found between PUFAs, *Lachnoclostridium*, and *Roseburia*. Additionally, *Phocaeicola* was positively correlated with acetic acid levels. Accordingly, DMB intake and nutritional treatment positively modify the intestinal microbiome in patients with cancer and dysgeusia, which might lead to a greater immunological response and better dietary intake.

## Figures and Tables

**Table 1:** Relative abundances of intestinal bacteria at the phylum level in malnourished patients with cancer and dysgeusia who received standard-dose DMB (150 mg), high-dose DMB (300 mg), or placebo for 3 months.

Phylum	DMB 150 mg		DMB 300 mg		Placebo		*p*-Value		
	Baseline	3 Months	Baseline	3 Months	Baseline	3 Months	Treatment (T)	Time (t)	T × t
*Actinobacteriota*	0.3 (0.1–0.7)	0.4 (0.1–0.4)	0.2 (0.03–0.7)	0.2 (0.07–0.5)	0.3 (0.01–0.8)	0.1 (0.03–0.6)	0.717	0.357	0.468
*Bacillota*	78.1 (70.6–94.1)	88.6 (76–95.1)	75.7 (49.3–86)	74.7 (67.7–80.6)	69.1 (13.1–88.1)	73.7 (50.3–91.1)	0.062	0.224	0.598
*Bacteroidota*	14.6 (1.4–24.5)	8.2 (1.9–20.7)	8.9 (3.1–41.4)	16 (3–26.4)	10.2 (0.008–18.1)	6.7 (0.1–32.4)	0.444	0.674	0.888
*Pseudomonadota*	3.8 (2.2–7.9)	2 (1.2–12)	7.2 (4.6–24)	8.1 (3.4–21.5)	16.8 (2.7–86.8)	15.9 (4.2–31.4)	0.043 *	0.253	0.366
*Tenericutes*	0.2 (0.01–0.3)	0.06 (0.01–0.2)	0.09 (0.01–0.2)	0.03 (0.008–0.2)	0.1 (0.006–0.4)	0.06 (0.01–0.2)	0.542	0.092	0.703
*Synergistetes*	0.1 (0.008–0.3)	0.08 (0.01–0.2)	0.08 (0.01–1.3)	0.2 (0.02–0.5)	0.2 (0.05–0.2)	0.1 (0.01–0.2)	0.282	0.397	0.876
*Verrucomicrobiota*	0.1 (0.006–0.9)	0.1 (0.01–1.5)	0.03 (0.007–1.3)	0.05 (0.01–0.2)	0.08 (0.07–1.2)	0.04 (0.01–2.4)	0.616	0.466	0.388
Shannon	3.3 (2.5–3.6)	3.3 (3.1–3.8)	3.3 (2.3–3.7)	3.3 (2.6–3.4)	3.3 (1.5–3.3)	3.0 (2.1–3.8)	0.150	0.879	0.745
Simpson	0.9 (0.8–0.9)	0.9 (0.9–1.0)	0.9 (0.8–0.9)	0.9 (0.9–0.9)	0.9 (0.5–0.9)	0.9 (0.8–1.0)	0.135	0.825	0.579
Chao1	435.2 (308.3–548.2)	388.7 (315.0–540.0)	404.7 (255.1–684.8)	422.6 (261.0–491.2)	394.7 (264.3–514.2)	415.6 (272.1–547.0)	0.367	0.139	0.202

The values are presented as medians and ranges. Based on the median test across time points within groups, * indicates significant differences (*p* < 0.05).

**Table 2:** Relative abundances of intestinal bacteria at the genus level in malnourished patients with cancer and dysgeusia who received standard-dose DMB (150 mg), high-dose DMB (300 mg), or placebo for 3 months.

Genus	DMB 150 mg		DMB 300 mg		Placebo		*p*-Value		
	Baseline	3 Months	Baseline	3 Months	Baseline	3 Months	Treatment (T)	Time (t)	T × t
*Faecalibacterium*	17.5 (5.7–34.1)	13.4 (12–24.2)	19.7 (4.6–28.8)	17.2 (4.6–28.8)	11.4 (0.01–35)	12.2 (0.05–46.3)	0.614	0.707	0.670
*Prevotella*	0.04 (0.01–10.8)	0.4 (0.01–15.8)	1.7 (0.02–30.9)	5.6 (0.1–23.8)	3.9 (0.01–15.5)	3.7 (0.1–27.9)	0.669	0.936	0.722
*Blautia*	4.2 (2.4–8.4)	4.9 (2.3–7.8)	4.7 (0.5–10.1)	3.9 (2.3–7.5)	2.9 (0.02–9.0)	2.9 (0.02–4.4)	0.382	0.236	0.828
*Anaerobutyricum*	4.0 (1.0–7.8)	2.4 (1.6–5.4)	2.6 (0.2–6.5)	2.3 (1.2–5.3)	1.9 (0.5–2.9)	3 (0.01–4.2)	0.369	0.848	0.126
*Dysosmobacter*	3.3 (0.8–4)	3.7 (0.7–6.3)	1.5 (0.5–7.1)	2.9 (0.3–9.3)	2.6 (1.3–3.8)	2.6 (0.02–6.2)	0.841	0.299	0.859
*Vescimonas*	3.3 (0.2–10.6)	3.3 (1.1–18.8)	2.6 (0.1–8.6)	1.3 (0.03–8.4)	6.0 (0.5–17.4)	2.8 (0.01–12.6)	0.450	0.977	0.130
*Roseburia*	2.8 (0.7–21.4)	1.7 (0.9–23.4)	2.4 (1.2–13.2)	3.6 (0.4–11.6)	1.6 (1.4–4.9)	3.2 (1.6–12.4)	0.830	0.506	0.547
*Sulcia*	2.8 (2.8–2.8)	1.1 (1.1–1.1)	0 (0–0)	0 (0–0)	0.3 (0.3–0.3)	0 (0–0)	1.0	1.0	1.0
*Bacteroides*	2.5 (0.4–9.4)	1.3 (0.7–4.8)	1.9 (0.8–14)	2.2 (0.6–8)	0.9 (0.3–15.5)	0.7 (0.02–7.7)	0.915	0.131	0.957
*Lachnospira*	2.1 (1.3–4)	2.5 (0.5–3.4)	2.5 (0.4–3.4)	1.3 (0.3–3.8)	1.1 (0.6–10.8)	1.8 (0.6–6.4)	0.576	0.459	0.874
*Clostridium*	1.8 (1.2–2.4)	1.9 (0.9–4.3)	2.1 (0.2–4.1)	1.3 (0.9–16.2)	1.2 (0.9–3.1)	1.4 (0.7–40.1)	0.614	0.182	0.513
*Coprococcus*	1.5 (1.0–3.6)	2.2 (0.8–2.8)	0.9 (0.06–5.0)	1.1 (0.2–2.2)	1.0 (0.2–2.7)	1.9 (0.5–2.9)	0.558	0.630	0.590
*Blattabacterium*	1.5 (1.5–1.5)	0.5 (0.5–0.5)	0 (0–0)	0 (0–0)	0.2 (0.2–0.2)	0 (0–0)	1.0	1.0	1.0
*Phascolarctobacterium*	1.4 (0.2–3)	1.9 (1.4–10.8)	2 (1.1–2.6)	1.6 (0.08–6.7)	0.03 (0.007–8.4)	0.01 (0.006–9.2)	0.966	0.206	0.586
*Mediterraneibacter*	1.3 (0.5–3.3)	0.8 (0.4–2.5)	1.6 (0.3–9)	1.5 (0.4–4.5)	0.7 (0.5–1.5)	0.8 (0.03–4.8)	0.379	0.514	0.350
*Dorea*	1.2 (0.9–2.9)	1.3 (0.8–2.8)	2.3 (0.3–4.3)	1.0 (0.07–10.2)	0.8 (0.06–1.9)	1.0 (0.09–1.4)	0.214	0.628	0.668
*Phocaeicola*	1.2 (0.3–5.2)	0.9 (0.09–2.8)	2.7 (0.3–4.0)	2.2 (0.6–4.2)	0.5 (0.3–0.7)	0.4 (0.4–0.5)	0.036 *	0.430	0.619
*Ruminococcus*	1.1 (0.4–22.9)	0.9 (0.7–19.2)	0.8 (0.1–5.3)	0.8 (0.3–1)	1.7 (0.2–5.3)	1.2 (0.008–3.2)	0.491	0.055	0.841
*Solibaculum*	1.1 (0.4–10.8)	3.4 * (0.8–7.8)	1.0 (0.07–6.7)	1.0 (0.08–6.4)	5.8 (0.03–8.0)	1.4 * (0.02–3.0)	0.782	0.172	0.046 *
*Herbinix*	1.0 (0.3–2.8)	1.6 (0.2–2.4)	0.6 (0.2–1.9)	0.8 (0.1–1.5)	1.1 (0.5–4.6)	1.5 (0.8–3.3)	0.403	0.740	0.685
*Lachnoclostridium*	0.9 (0.5–2.3)	0.6 (0.4–3.0)	0.8 (0.2–1.9)	0.6 (0.3–3.5)	0.6 (0.01–0.8)	0.6 (0.2–2.8)	0.487	0.103	0.934
*Anaerostipes*	0.9 (0.3–6.7)	1.0 (0.4–7.2)	0.8 (0.3–4.0)	0.8 (0.3–1.2)	0.5 (0.2–6.0)	1.0 (0.01–5.7)	0.801	0.617	0.806
*Escherichia*	0.8 (0.2–2.7)	0.3 (0.1–2.9)	1.3 (0.1–14.0)	1.7 (0.2–5.0)	1.1 (0.3–19.3)	3.4 (0.3–9.2)	0.012 *	0.291	0.756

The values are presented as medians and ranges. Based on the median test across time points within groups, * indicates significant differences (*p* < 0.05).

**Table 3:** Relative abundances of intestinal bacteria at the species level in malnourished patients with cancer and dysgeusia who received standard-dose DMB (150 mg), high-dose DMB (300 mg), or placebo for 3 months.

Species	DMB 150 mg		DMB 300 mg		Placebo		*p*-Value		
	Baseline	3 Months	Baseline	3 Months	Baseline	3 Months	Treatment (T)	Time (t)	T × t
*Bacteroides caccae*	0.4 (0.04–0.9)	0.2 (0.01–1.0)	0.2 (0.07–1.1)	0.08 (0.04–0.3)	0.03 (0.02–0.4)	0.02 (0.02–1.0)	0.985	0.197	0.084
*Bacteroides stercoris*	0.2 (0.03–0.4)	0.2 (0.01–1.0)	0.2 (0.01–1.5)	0.5 (0.05–0.8)	0.1 (0.08–0.1)	0.3 (0.03–0.5)	0.587	0.608	0.713
*Bacteroides thetaiotaomicron*	0.5 (0.06–1.0)	0.3 (0.03–0.8)	0.07 (0.04–0.8)	0.1 (0.06–0.5)	0.1 (0.04–5.3)	0.2 (0.09–2.1)	0.591	0.197	0.475
*Bacteroides uniformis*	0.5 (0.03–2.7)	0.4 (0.08–2.1)	0.3 (0.03–1.9)	0.6 (0.03–2.1)	0.2 (0.06–0.4)	0.2 (0.1–0.5)	0.552	0.554	0.655
*Bacteroides* sp. PHL 2737	1.5 (0.8–2.2)	0.3 * (0.3–0.3)	2.1 (0.2–4.0)	0.4 * (0.2–2.2)	0.4 (0.3–0.4)	0.2 (0.2–0.2)	0.469	<0.001 *	<0.001 *
*Anaerobutyricum hallii*	4.0 (1.0–7.8)	2.5 (1.6–5.4)	2.6 (0.2–6.6)	2.3 (1.2–5.4)	1.9 (0.5–2.9)	3.0 (0.01–4.3)	0.366	0.853	0.128
*Blautia argi*	0.3 (0.2–0.9)	0.2 (0.2–0.5)	0.3 (0.04–0.9)	0.2 (0.1–0.4)	0.2 (0.1–0.3)	0.2 (0.1–0.3)	0.136	0.287	0.688
*Blautia liquoris*	1.0 (0.6–5.8)	1.0 (0.5–3.9)	1.0 (0.2–1.6)	1.2 (0.1–1.7)	0.4 (0.2–3.0)	0.9 (0.6–2.9)	0.624	0.606	0.364
*Blautia massiliensis*	0.6 (0.3–2.5)	0.9 (0.4–2.5)	1.4 (0.05–3.8)	1.0 (0.2–2.3)	0.5 (0.01–1.2)	0.4 (0.1–0.7)	0.194	0.265	0.426
*Blautia obeum*	0.4 (0.04–1.1)	0.07 (0.05–0.9)	0.1 (0.08–2.0)	0.04 (0.03–0.3)	0.1 (0.02–0.5)	0.1 (0.06–0.3)	0.620	0.269	0.521
*Blautia pseudococcoides*	0.1 (0.09–0.2)	0.2 (0.1–0.3)	0.2 (0.04–0.3)	0.1 (0.08–0.2)	0.1 (0.07–0.2)	0.09 (0.05–0.2)	0.199	0.945	0.801
*Blautia* sp. SC05B48	1.0 (0.4–1.9)	0.8 (0.4–2.9)	1.1 (0.1–3.8)	1.0 (0.5–4.4)	1.2 (0.5–8.3)	0.8 (0.3–2.9)	0.720	0.283	0.278
*Lachnospira eligens*	2.1 (1.3–4.0)	2.5 (0.6–3.4)	2.5 (0.4–3.4)	1.4 (0.3–3.8)	1.1 (0.6–10.8)	1.8 (0.6–6.5)	0.576	0.462	0.873
*Roseburia hominis*	2.5 (0.6–19.3)	1.7 (0.8–17.3)	2.0 (0.2–4.8)	2.3 (0.1–6.9)	1.4 (1.1–3.9)	2.7 (1.3–4.2)	0.561	0.975	0.356
*Roseburia intestinalis*	0.3 (0.07–2.0)	0.5 (0.1–5.5)	0.6 (0.03–8.8)	1.2 (0.2–4.5)	0.3 (0.1 -1.0)	0.5 (0.3–5.8)	0.533	0.421	0.494
*Roseburia* sp. NSJ-69	0.01 (0.007–0.3)	0.04 (0.02–0.8)	0.1 (0.01–0.3)	0.1 (0.01–0.5)	0.03 (0.02–0.09)	0.1 (0.08–2.4)	0.423	0.172	0.659
*Faecalibacterium prausnitzii*	17.7 (5.8–34.4)	13.9 (12.1–24.6)	19.8 (4.6–28.9)	17.3 (4.6–29.0)	11.5 (0.01–35.7)	12.3 (0.07–46.7)	0.620	0.712	0.678
*Vescimonas coprocola*	2.3 (0.06–7.7)	1.6 (0.5–9.6)	1.6 (0.1–2.6)	0.9 (0.03–3.3)	3.9 (0.5–13.0)	2.2 * (0.02–8.9)	0.245	0.889	0.049 *
*Vescimonas fastidiosa*	1.1 (0.2–9.0)	2.5 (0.6–9.5)	1.0 (0.02–6.4)	0.7 (0.04–6.1)	2.2 (1.9–4.6)	1.3 (0.01–3.8)	0.719	0.881	0.563
*Dysosmobacter marseille*	0.5 (0.3–1.0)	0.8 (0.2–2.2)	0.2 (0.03–1.5)	0.4 (0.03–1.3)	0.8 (0.5–0.9)	0.9 (0.4–1.1)	0.421	0.405	0.416
*Dysosmobacter welbionis*	2.5 (0.6–3.4)	3.1 (0.5–4.6)	1.3 (0.5–7.1)	2.3 (0.3–9.2)	1.8 (0.6–2.9)	1.9 (0.3–5.2)	0.897	0.207	0.988

The values are presented as medians and ranges. Based on the median test across time points within groups, * indicates significant differences between baseline and 3 months (*p* < 0.05).

**Table 4:** Plasma short-chain fatty acids in malnourished patients with cancer and dysgeusia who received standard-dose DMB (150 mg), high-dose DMB (300 mg), or placebo for 3 months.

Short-Chain Fatty Acids	DMB 150 mg		DMB 300 mg		Placebo		*p*-Value		
	Baseline	3 Months	Baseline	3 Months	Baseline	3 Months	Treatment (T)	Time (t)	T × t
Acetic acid (µmol/L)	12.8 ± 6.4	26.7 ± 6.7 *	36.6 ± 7.0	31.7 ± 7.3	25.0 ± 7.8	15.0 ± 8.2 *	0.082	0.032 *	0.027 *
Propionic acid (µmol/L)	0.8 ± 0.6	2.2 ± 0.9	1.5 ± 0.6	0.6 ± 1.0	1.9 ± 0.7	2.6 ± 1.1	0.357	0.420	0.559
Isobutyric acid (µmol/L)	0.3 ± 0.1	0.4 ± 0.1	0.3 ± 0.1	0.3 ± 0.1	0.4 ± 0.1	0.4 ± 0.1	0.993	0.893	0.955
Butyric acid (µmol/L)	0.9 ± 0.3	1.3 ± 0.3	0.9 ± 0.3	0.8 ± 0.4	1.1 ± 0.3	1.3 ± 0.4	0.698	0.414	0.591
Isovaleric acid (µmol/L)	0.2 ± 0.1	0.3 ± 0.1	0.2 ± 0.1	0.3 ± 0.1	0.2 ± 0.1	0.4 ± 0.1	0.865	0.991	0.603
Valeric acid (µmol/L)	1.0 ± 0.8	2.2 ± 0.9	1.0 ± 1.5	0.7 ± 1.0	1.0 ± 1.9	2.7 ± 1.1	0.573	0.559	0.878

The values are presented as the means and standard deviations. Based on the median test across time points within groups, * indicates significant differences (*p* < 0.05).

## Data Availability

A reasonable request should be made to the corresponding author for access to the datasets used and/or analyzed in the current study.

## Supplementary Materials

The following supporting information can be downloaded at https://www.mdpi.com/article/10.3390/nu17020246/s1: Table S1: Nutritional composition of the food supplement enriched in miraculin (DMB) and placebo; Table S2: Summary of key biochemical parameters of the study population; Table S3: Cancer types and chemotherapy characteristics of the study population.
