# Supplementary material for: Effect of a Novel Food Rich in Miraculin on the Intestinal Microbiome of Malnourished Patients with Cancer and Dysgeusia
Source: Nutrients. 2025 Jan 10;17(2):246. doi: 10.3390/nu17020246 (PMC11767858; doi:10.3390/nu17020246)
Supplement: Supplementary file 1 [file nutrients-17-00246-s001.zip › nutrients-3382216-supplementary.pdf]

Table S1. Nutritional composition of the food supplement enriched in miraculin (DMB) and placebo

		Standard dose of DMB (150 mg DMB + 150 mg strawberry freeze-dried)	High dose of DMB (300 mg DMB)	Placebo (300 mg strawberry freeze-dried)
Energy	kcal	0.99	1	0.97
Carbohydrates	mg	194	234	154
Sugars	mg	156	162	150
Fiber	mg	26	6	46
Proteins	mg	20	15	24
Lipids	mg	9	5	12
Saturated fatty acids	mg	2	2	1
Sodium chloride	mg	0.1	0.1	0.03
Humidity	mg	4	4	5
Ash	mg	12	14	15
Miraculin	mg	2.8	5.6	0

Nutritional composition provided by Medicinal Gardens, S.L.

Table S2. Summary of key blood and biochemical parameters of patients included in the CLINMIR study

Parameters	DMB 150 mg				DMB 300 mg				Placebo				p-value		
	Baseline	1 month	2 months	3 months	Baseline	1 month	2 months	3 months	Baseline	1 month	2 months	3 months	Treatment (T)	Time (t)	T x t
Leukocytes ($\times 10^3$/uL)	8.6 ± 10.4	6.6 ± 3.5	7.5 ± 4.5	6.0 ± 3.6	8.1 ± 6.4	4.9 ± 1.9	5.1 ± 2.0	5.2 ± 2.5	9.5 ± 7.4	8.4 ± 4.3	11.8 ± 8.1	7.4 ± 5.0	0.285	0.199	0.812
Red blood cells ($\times 10^6$/uL)	4.1 ± 0.6	4.1 ± 0.6	4.1 ± 0.5	3.9 ± 0.7	4.3 ± 0.8	4.2 ± 1.0	4.2 ± 0.7	4.3 ± 0.6	3.8 ± 0.6	3.8 ± 0.5	3.9 ± 0.5	3.9 ± 0.5	0.434	0.71	0.831
Hemoglobin (g/dL)	12.6 ± 1.7	12.7 ± 1.4	12.9 ± 1.0	12.4 ± 1.8	13.1 ± 2.1	13.0 ± 2.1	13.2 ± 1.8	13.3 ± 1.4	11.7 ± 1.3	11.6 ± 1.3	11.9 ± 0.8	11.7 ± 1.3	0.182	0.556	0.947
Hematocrit (%)	39.0 ± 4.4	39.3 ± 3.6	34.8 ± 12.5	37.9 ± 4.7	40.6 ± 6.5	40.1 ± 7.2	40.9 ± 5.3	41.3 ± 4.1	36.4 ± 3.9	36.7 ± 3.7	37.7 ± 2.4	37.0 ± 4.2	0.277	0.971	0.81
Mean Corpuscular Volume (fL)	96.1 ± 6.9	97.4 ± 8.4	97.8 ± 8.9	97.8 ± 8.4	95.4 ± 6.0	97.0 ± 6.8	97.3 ± 6.1	96.6 ± 8.2	97.6 ± 6.9	98.3 ± 6.7	97.2 ± 6.8	96.4 ± 5.7	0.977	0.147	0.454
Mean Corpuscular Hemoglobin (g/dL)	31.1 ± 2.4	31.5 ± 3.1	31.9 ± 3.2	31.9 ± 2.8	30.7 ± 2.0	31.5 ± 2.8	31.3 ± 2.2	31.1 ± 2.5	31.2 ± 2.7	31.0 ± 2.2	30.7 ± 2.5	30.6 ± 2.2	0.856	0.498	0.096
Mean corpuscular hemoglobin concentration (g/dL)	32.3 ± 0.8	32.3 ± 0.8	32.5 ± 1.1	32.6 ± 1.1	32.2 ± 0.7	32.5 ± 0.8	32.2 ± 0.5	32.2 ± 0.6	32.0 ± 1.1	31.5 ± 0.8	31.6 ± 0.6	31.7 ± 0.6	0.126	0.973	0.386
Platelet count ($\times 10^3$/uL)	260.6 ± 123.3	223.6 ± 80.7	229.6 ± 101.2	235.3 ± 106.4	207.2 ± 81.2	200.3 ± 83.0	227.6 ± 85.6	246.5 ± 129.3	232.9 ± 136.5	221.7 ± 59.0	219.0 ± 65.6	218.4 ± 66.9	0.923	0.753	0.729
Creatinine (mg/dL)	0.8 ± 0.3	0.8 ± 0.3	0.9 ± 0.3	0.8 ± 0.3	0.7 ± 0.2	0.7 ± 0.2	0.7 ± 0.2	0.7 ± 0.3	1.0 ± 1.0	0.7 ± 0.1	0.7 ± 0.1	0.8 ± 0.2	0.033	0.329	0.054
Uric acid (mg/dL)	4.25 ± 1.27	4.6 ± 1.8	4.9 ± 2.1	4.1 ± 1.5	4.4 ± 1.2	4.6 ± 0.8	4.6 ± 1.0	4.6 ± 1.2	5.1 ± 2.0	4.3 ± 1.1	4.8 ± 1.0	5.0 ± 1.3	0.057	0.16	0.066
Alanine transaminase (UI/L)	34.2 ± 18.0	24.3 ± 12.9	27.9 ± 15.0	23.9 ± 12.8	26.6 ± 13.3	25.1 ± 18.9	31.2 ± 24.6	29.5 ± 13.8	32.1 ± 11.6	32.7 ± 14.5	36.4 ± 16.5	37.7 ± 18.0	0.051	0.221	0.057
Aspartate aminotransferase (UI/L)	31.6 ± 12.61	29.5 ± 18.7	27.1 ± 18.4	26.4 ± 12.3	29.4 ± 17.6	31.0 ± 22.1	35.9 ± 30.0	31.7 ± 21.6	26.8 ± 15.2	26.0 ± 10.8	28.3 ± 20.3	28.1 ± 14.2	0.985	0.778	0.903
Lactate dehydrogenase (UI/L)	225.6 ± 25.7	230.8 ± 43.4	238.8 ± 27.9	229.3 ± 23.8	291.8 ± 154.2	236.1 ± 77.9	272.1 ± 109.2	240.1 ± 117.9	520.7 ± 841.9	258.0 ± 65.7	262.0 ± 53.6	248.1 ± 60.6	0.865	0.267	0.996
Alkaline phosphatase (UI/L)	82.6 ± 27.3	79.2 ± 20.2	69.8 ± 9.7	72.1 ± 10.4	149.6 ± 138.7	145.6 ± 172.6	167.8 ± 199.7	89.0 ± 31.0	146.8 ± 76.0	128.1 ± 43.1	131.1 ± 46.5	126.5 ± 40.8	0.934	0.264	0.982
Gamma-glutamyl transferase (UI/L)	39.1 ± 20.3	35.3 ± 19.2	31.0 ± 17.1	34.1 ± 18.7	168.3 ± 389.0	235.0 ± 523.7	250.0 ± 544.6	43.2 ± 29.4	142.6 ± 157.8	92.3 ± 92.8	117.7 ± 129.2	116.1 ± 122.1	0.727	0.537	0.748
Albumin (g/dL)	4.4 ± 0.2	4.2 ± 0.2	4.3 ± 0.2	4.1 ± 0.5	4.2 ± 0.3	4.1 ± 0.2	4.2 ± 0.2	4.3 ± 0.2	4.3 ± 0.4	4.2 ± 0.3	4.2 ± 0.2	4.3 ± 0.2	0.062	0.595	0.114
CRP (mg/dL)	0.5 ± 0.9	0.2 ± 0.2	0.3 ± 0.5	0.9 ± 1.7	0.8 ± 0.5	0.8 ± 1.0	1.0 ± 1.5	2.3 ± 4.0	0.7 ± 0.8	1.1 ± 1.2	1.4 ± 2.4	0.9 ± 1.5	0.523	0.467	0.314
Zinc (µmol/L)	11.8 ± 2.2	-	-	11.0 ± 2.5	11.5 ± 1.7	-	-	12.1 ± 1.7	11.7 ± 3.3	-	-	1124.4 ± 155.3	0.922	0.738	0.666

Values are expressed as mean ± standard deviation.

Table S3. Cancer types and chemotherapy characteristics of patients included in the CLINMIR study

Variables		DMB 150 mg	DMB 300 mg	Placebo	p-value
Sex	Female (%)	70	45.5	60	0.517
	Male (%)	30	54.5	40	
Age	years	59.9 ± 15.1	58.9 ± 4.9	61.3 ± 11.2	0.891
Weight	kg	61.4 ± 11.1	62.0 ± 14.1	62.6 ± 10.7	0.941
Weight lost in last 6 months	%	7.5 ± 6.0	8.7 ± 7.1	7.2 ± 8.0	0.868
BMI	kg/m^2	21.9 ± 3.6	22.0 ± 3.3	22.9 ± 3.4	0.737
Type of cancer					
Head and neck	%	0.00	9.10	0.00	
Colorectal	%	30.00	27.30	20.00	
Esophagus	%	10.00	0.00	10.00	
Stomach	%	0.00	9.10	10.00	
Liver	%	0.00	9.10	10.00	
Breast	%	10.00	18.20	10.00	0.895
Neuroendocrine	%	10.00	0.00	0.00	
Ovary	%	10.00	18.20	0.00	
Pancreas	%	10.00	9.10	10.00	
Lung	%	10.00	0.00	10.00	
Others	%	10.00	0.00	20.00	
Chemotherapy	%	100.0	100.0	100.0	1
Radiotherapy	%	20.00	12.50	0.00	0.594
Smoking status (yes)	%	30.00	27.30	40.00	0.605
Alcohol use (yes)	%	20.00	27.30	10.00	0.393
Dental Disease (yes)	%	0.00	0.00	0.00	1

BMI, body mass index. Values are expressed as mean ± standard deviation
